# The Ultrastructures and Mechanical Properties of the Descement’s Membrane in Fuchs Endothelial Corneal Dystrophy

**DOI:** 10.1038/srep23096

**Published:** 2016-03-16

**Authors:** Dan Xia, Shuai Zhang, Esben Nielsen, Anders Ramløv Ivarsen, Chunyong Liang, Qiang Li, Karen Thomsen, Jesper Østergaard Hjortdal, Mingdong Dong

**Affiliations:** 1 Research Institute for Energy Equipment Materials, Tianjin Key Laboratory of Materials Laminating Fabrication and Interface Control Technology, Hebei University of Technology, Tianjin 300130, China; 2 The Interdisciplinary Nanoscience Center, Aarhus University, Aarhus 8000, Denmark; 3 Department of Ophthalmology, Aarhus University Hospital, Aarhus 8000, Denmark

## Abstract

Fuchs endothelial corneal dystrophy (FECD), is the most common corneal endothelial dystrophy, and contributes up to 50% of all corneal transplantations performed in developed countries. FECD develops in Descemet’s membrane (DM) and possibly alters the mechanical properties and internal structures in this basal lamina. In this work, the morphology and mechanical properties of FECD-DMs are studied by transmission electron microscopy (TEM) and quantitative dynamic atomic force microscopy (QD-AFM) at nano scale. Pathological wide-space collagens that are typical of FECD display different mechanical properties in that they are softer than the remaining tissue both for dehydrated- and fully hydrated samples. Additionally, the hydration level has major influence on the mechanical properties. These findings could help to further understand the structural changes in FECD, and possibly be useful for further characterization of the disease, the diagnosis and assessment or even pathologic analysis.

Fuchs’ endothelial corneal dystrophy (FECD) is a common corneal dystrophy, which was first described as ‘dystrophia epithelialis corneae’ in 1910 by Ernst Fuchs^1^. This slowly progressive, bilateral corneal dystrophy has a predilection for women at a ratio of 2.5–3:1, and an early-onset and late-onset variant has been identified^2^,^3^,^4^,^5^,^6^. Early-onset FECD is quite rare and generally regarded as an autosomal dominant disorder with a Mendelian inheritance pattern^7^,^8^. Late-onset FECD however, is autosomal dominant with variable penetrance, which is the most common disorder of the corneal endothelium. FECD shows prevalence in residents all over the world (9.2% prevalence over 55 years old, Iceland^9^; 6.7% prevalence over 50 years old Chinese Singaporean; 3.7% prevalence over 50 years old Japanese population^10^). It is characterized by thickening of Descement’s membrane (DM), deposition of focal excrescences, termed “guttae”, and progressive loss of corneal endothelial cells^11^,^12^. The cornea is normally maintained in a relatively dehydrated state (deturgescence^11^) by the endothelial cells which pumps out fluid of the cornea. This keeps the cornea transparent. However, the number of endothelial cells will gradually decline as FECD progresses, which will inevitably lead to influx of water and corneal edema^3^. Vision is severely impaired due to loss of corneal transparency, and in late stages of the disease painful epithelial bullae can form^13^.

The DM is formed in foetal life and the endothelial cells continuously add a small amount of material throughout life-thus making the DM increase slightly in thickness and takes form of a basement membrane^14^. Since FECD primarily affects the DM and endothelial cell layers^15^, the study of the pathologic ultrastructure and mechanical properties of DM is critical for further understanding the pathogenesis of FECD. Although our knowledge of FECD has expanded greatly in recent years, the precise biomechanical events resulting by FECD is still unknown. Here, the ultrastructure and mechanical properties of FECD-DMs are compared to normal DMs by both transmission electron microscopy (TEM) and quantitative dynamic atomic force microscopy (QD-AFM)^16^. The TEM results show that FECD-DMs are thicker than in controls and has various fibrillar structures. QD-AFM studies show that DMs in air are very stiff with the stiffness in the GPa range, with the wide-spaced collagen being softer than the remaining FECD-DM tissue. The mechanical properties of DMs in physiological buffer have stiffness in the MPa range. The wide-spaced collagen is softer as it is in dry condition, and it has increased swelling capacity than the rest parts of the tissue. These findings reveal that the mechanical properties of FECD-DMs are significantly changed compared with DM from controls. These mechanical changes may be one contributing factor in the pathogenesis of FECD.

## Results and Discussion

**Figure 1:**
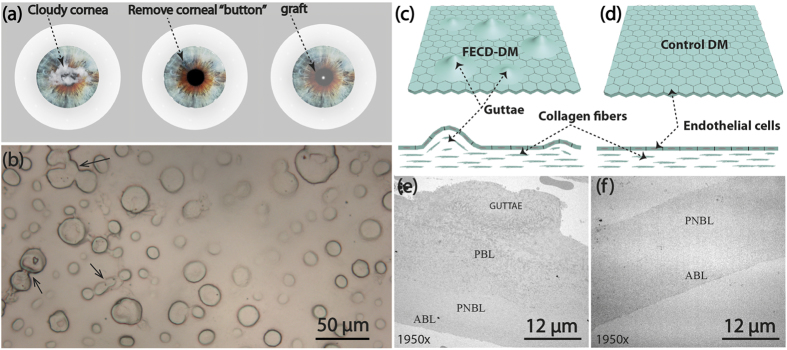
(**a**) Scheme of the corneal transplant procedure; (**b**) Optical image of the FECD-DM; Scheme of (**c**) the FECD-DM with different size guttaes and (**d**) the control DM (top panel, side view; bottom panel, cross-section view); TEM images of the (**e**) FECD-DM and (**f**) control DM.

FECD-DMs were obtained from posterior lamellar corneal transplant surgery, which replaces the diseased corneal endothelium and DM with donor corneal tissue (the graft) (Fig. 1a). The removed DM contained guttae with different sizes varied from 5 μm up to 50 μm as revealed by the optical microscopy (Fig. 1b). The characteristic feature of FECD, guttae, initially deposits at the centre, and then spread toward the periphery of the basement membrane. The guttae coalesce, which suggest growth (Indicated by the black arrow in Fig. 1b). The representative cross-sections of the FECD-DM/endothelium and control DM/endothelium layers are schemed in Fig. 1c,d, respectively. The formation of guttae on FECD-DM may lead to light scatter, compared to a normal cornea, which may severely reduce vision. Another hallmark feature of FECD is thickening DM, which is confirmed by TEM images (Fig. 1e,f). It is apparent that the FECD-DM is thicker than normal DM even without protrusion of the guttae. By analysing the substructure of the DM, an additional posterior banded layer (PBL) is found. The individual layers of normal DM and FECD-DM are labelled as shown in Fig. 1e,f, respectively. In control samples, there are mainly two layers, the anterior banded layer (ABL) and the posterior non-banded layer (PNBL). The ABL is mainly composed of thin and short filaments, while PNBL is cross-linked to longer filaments with non-striated, non-lamellar amorphous material. The thickness of the healthy ABL is almost unchanged with aging^17^. However, the thickness of PNBL increases with a velocity of approximately 0.1 μm/year gradually^18^,^19^. Further structural details on the layer in control DMs can be found in the Supporting Information, Figure S1. It shows, that the ABL is 4.6 ± 0.4 μm while the PNBL is 11.8 ± 0.4 μm. The PBL of FECD-DM^15^,^18^ contains a dense fibrous component and widely spaced collagen. The fibrils arrange in a disorganized fashion and contribute to the formation of guttae (Fig. 1e)^20^. Hence, the FECD-DM is much thicker (21.9 ± 2.4 μm) than the normal DM (18.8 ± 0.2 μm), and even thicker (38.9 ± 1.7 μm) when companied with the guttae. The ABL in FECD-DM is approximate 3 μm, which is comparable to the thickness of normal DM. However, the PNBL in FECD-DM is much thinner than that in normal DM, and may even disappear in some cases^15^,^17^.

**Figure 2:**
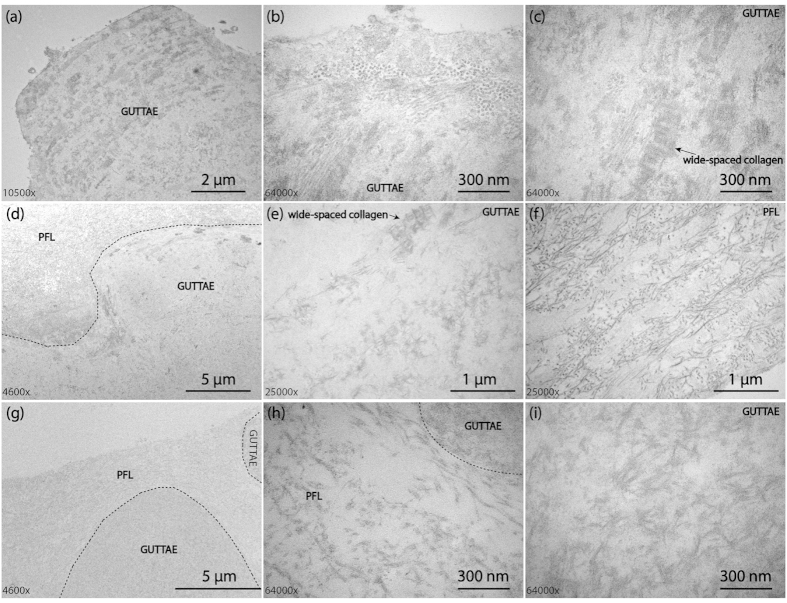
TEM images of three types FECD-DMs obtained from corneal transplant surgery. (**a–c**) Type I FECD-DM; (**a**) Low magnification image; High magnification image of guttae (**b**) at the edge and (**c**) at the center; (**d–f**) Type II FECD-DM with an additional PFL; (**d)** The image on the boundary between PFL and guttae; (**e**) The zoom in image on the guttae; (**f**) The zoom in image on the PFL layer; (**g–i**) Type III FECD-DM with two layers of guttae; (**g**) The two layers of guttae separated with each other by the PFL layer; (**h**) The image of porous PFL near the guttae; (**i**) The image on the guttae. The black dash lines in (**d**), (**g**,**h**) indicate the boundary.

Healthy DM is composed of different types of collagen (type IV^21^,^22^,^23^, VIII, XII, XVIII), glycoproteins (fibronectin^24^, laminin^23^, osteonectin) and proteoglycans (versican)^25^. However, FECD alters the composition of DM by increasing the deposition of collagen IV and VIII, fibronectin, and laminin on the posterior surface leading to a thickening of the DM^20^,^26^. Generally, three types of FECD-DMs were found in this study as shown in Fig. 2. Type I FECD-DM has the three typical layers: ABL, PNBL and PBL (Figs 1e and 2a–c, Supporting Information Figure S2a), which contains abnormally deposited collagen (wide-spaced collagen, *etc*.), the classic posterior excrescences, guttae^27^, occasional 10–20 nm fibrils, and amorphous substance. Type II FECD-DM (Fig. 2d–f) has an additional posterior fibrillar layer (PFL), a loose matrix of collagen with a fibril diameter of 20–40 nm^28^, compared to type I FECD-DM. The overview image of type II FECD-DM is shown in Supporting Information Figure S2b, in which guttae are buried into the fourth PFL. Type III FECD-DM forms two layers of guttae (Fig. 2g–i); one is buried in PFL as type II FECD-DM, while the other layer protrude as the type I FECD-DM (Supporting Information Figure S2c).

Collagen VIII has been identified to comprise a large part of the abnormally secreted posterior collagenous layer of DM, where the endothelial guttae form^29^,^30^. The alteration of the expression and deposition of collagen VIII may be relevant to the pathological response of the endothelium to ageing and trauma. One of the major forms of collagen VIII in the posterior collagenous layer is the wide-spaced collagen, which distributes on the whole PBL including the guttae area (Supporting Information Figure S2a). Figure 2a shows the morphology of one guttae on FECD-DM, consisting of wide-spaced collagen and other fibrillar collagenous structures. Interestingly, these fibrillar structures have a parallel orientation along the inner corneal surface. High magnification (Fig. 2b) shows the vertical fibrillar structures near the edge, which further indicates the parallel growth of guttae. The wide-spaced collagen can also be observed in high magnification images, as shown in Fig. 2c indicated by the black arrow. The morphologies of different type I FECD-DMs with different guttae size are shown in Supporting Information Figure S3, in which the fusiform wide-spaced collagen cross-linked with several smaller diameter collagen fibrils can be clearly seen. Figure 2d shows the morphology of type II FECD-DMs. A black dashed line indicates the boundary between the guttae and the PFL. High magnification image of a guttae (Fig. 2e) shows the ultrastructure of wide-spaced collagen with an orientation of a star-like matrix. The perfection of the star-like shape depends on the angle at which it is sectioned. From the observation of the ultrastructure, that wide-spaced collagen is structured by cross-linking of the star-like patterned fibers forming the fusiform bundles. The morphology of PFL is shown in Fig. 2f, where a loose matrix of collagen with the fiber size of 24.5 ± 2.9 nm can be clearly seen. In type III FECD-DM, PFL covers one layer of guttae and is adjacent to another layer of guttae (Fig. 2g and Supporting Information Figure S2c). Unlike the PFL of type II FECD-DM, this fibrillar layer is much looser and contains vacuoles (Fig. 2h). Also, the fusiform guttae structure is hardly seen in type III FECD-DM. Only few fusiform wide-spaced collagen structures are found on the boundary between the PFL and the outer layer of guttae. The dominant structures on guttae of type III FECD-DM are the star-like structure, shown in Fig. 2i.

**Figure 3:**
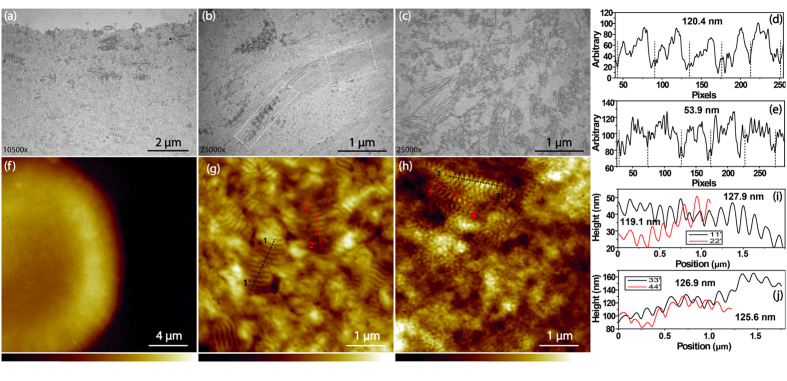
The TEM and AFM images of the wide-spaced collagen of FECD-DMs. (**a**) The wide-spaced collagen distributed on the posterior of the DM; (**b**) Different types of collagen in the FECD-DMs; (**c**) The morphology formed by the wide-spaced collagen together with other fibrillar collagen structures. (**d,e**) The line profiles of two types of collagens labelled in two white dash boxes in (**b**), which indicate different periodicities of different collagen types; (**f**) AFM image of a guttae; (**g**) The zoom in image on the guttea in (**f**); (**h**) AFM image near the guttae, the Z range in f is from 0 μm to 3.0 μm while that of in g and h are from 0 nm to 164.6 nm; (**i,j**) The line profiles of four color lines in (**g**,**h**), respectively.

Since the wide-spaced collagen exists in all types of FECD-DMs, more attention was addressed to this finding, and its ultrastructure was further studied. Figure 3a shows the fusiform wide-spaced collagen appearing on the surface layer of guttae. Higher magnification (Fig. 3b) shows that there are at least two types of collagen: wide-spaced collagen and one with smaller diameter, indicated by the white dashed squares. The line profiles drawn on each of the collagens confined in two white dashed boxes are shown in Fig. 3d,e. It demonstrates that the micro-periodicity of the wide-spaced collagen is 118.9 ± 5.9 nm, while the periodicity of the smaller collagen is 52.8 ± 2.1 nm. In addition, the FECD-DM morphology also shows the wide-spaced collagen cross-linked with the smaller diameter collagen (Fig. 3c). As TEM shows the cross-section structure of the sample, the corresponding surface structure of the FECD-DM could also be examined by AFM (Fig. 3f). As the guttae are much higher than the surface, the contrast only shows the guttae. Figure 3g, the zoomed in image from the white dash square in Fig. 3f, shows many wide-spaced collagens with different orientations, and the micro-periodicity can be clearly seen. The wide-spaced collagens are not only deposited on the guttae surface but also on other spots of PBL, as shown in Supporting Information Figure S4a. Combining Fig. 3f and S4a, one can see that FECD alters the morphology of the whole DM and the changed ultrastructures on the whole PBL including guttae are similar. The wide-spaced collagen cross-linked with other fibrillar collagen is also found by AFM (Fig. 3h). The overall image of the guttae and the zoom in image are shown in Supporting Information Figure S4b,c. The line profiles drawn on different collagens in Fig. 3g,h show the micro-periodicity of the wide-spaced collagen is also around 124.5 ± 5.5 nm, which is almost consistent with the TEM results (Fig. 3d).

**Figure 4:**
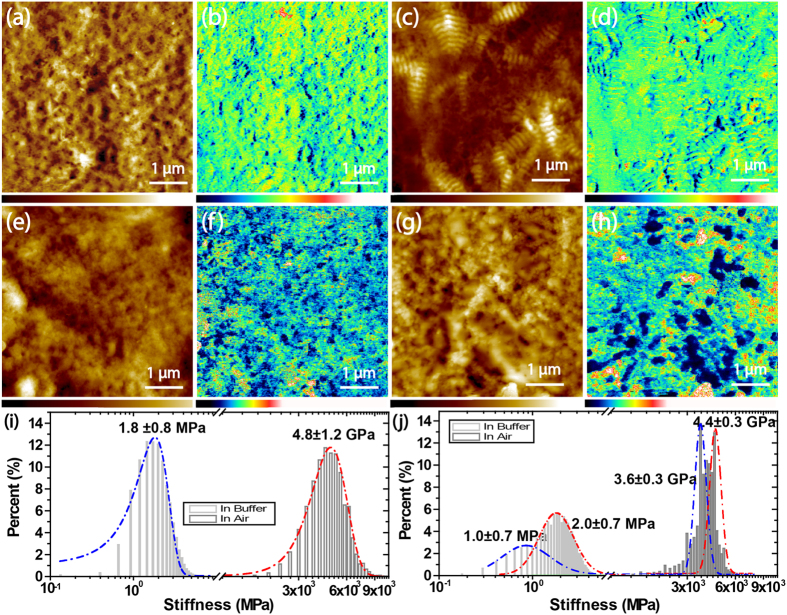
The comparison between the control and FECD-DMs. (**a,c,e,g**) The morphology images; (**b,d,f,h**) The stiffness maps; (**a,b**) The control sample in air; (**c**,**d**) The FECD-DM in air; (**e,f**) The control sample in physiological buffer; (**g,h**) The FECD sample in physiological buffer; The Z ranges in (**a,c**) are from 0 nm to 121.3 nm while that of in (**e,g**) are from 0 nm to 235.9 nm; The Z ranges in (**b,d**) are from 0 GPa to 17.8 GPa while that of in (**f,h**) are from 0 MPa to 4.8 MPa; The corresponding stiffness distribution of (**i**) the control and (**j**) FECD-DM in air and in physiological buffer, respectively.

The morphology change is normally related to changes in the mechanical properties, which may lead to dysfunction of the cornea. AFM has been proved able to characterize the mechanical properties of the cornea tissue^31^,^32^,^33^,^34^,^35^. To further understand the mechanism of FECD, the mechanical properties of the control/FECD-DMs were investigated by QD-AFM. Figure 4a–d shows the morphologies and the mechanical properties of the control/FECD-DMs under ambient air-dry condition. The morphology of the control sample shows porous homogenous structure (Fig. 4a) while the FECD sample shows with different sizes of wide-spaced collagen structures (Fig. 4c). The corresponding mechanical maps of these two samples are shown in Fig. 4b,d, respectively. The colour bars underneath Fig. 4b,d show that the red colour indicates high stiffness, while the blue colour indicates low stiffness. It is clearly that the stiffness of the control sample is almost homogenous, although some soft areas appear due to the porous structure of the control. However, for the FECD sample, it is apparent that the wide-spaced collagen is softer than the remaining structure in FECD-DM. The *in situ* stiffness distributions derived from the stiffness map show two distinguishing peaks located at 3.6 ± 0.3 GPa and 4.4 ± 0.3 GPa, respectively (Fig. 4j). Compared with the stiffness of the control sample (4.8 ± 1.2 GPa) (Fig. 4e), the wide-spaced collagens soften the FECD-DM, while the stiffness of remaining structure is almost the same as the control one.

The relative dehydrated state of the cornea depends on the active transport of ions by the endothelium. This in turn generates a swelling pressure as negatively charged side chains on glycosaminoglycans are forced into proximity^3^. As mentioned in our previous study, the cornea has an innate tendency to imbibe fluid and a swelling tendency exceeding any other connective tissue^35^. The cornea stroma and DM will swell if the endothelial layer is dysfunctional. Figure 4f–i shows the structure and mechanical properties of the control- and FECD-DMs in physiological buffer. Again, the control sample shows porous homogenous structure (Fig. 4f). The corresponding stiffness map (Fig. 4g) and *in situ* stiffness distribution (Fig. 4e) also demonstrates homogenous mechanical property with the stiffness of 1.8 ± 0.8 MPa. However, the structure of FECD-DM under physiological buffer is heterogeneous, the same as the result in ambient condition (Fig. 4h). In addition, it also shows that the wide-spaced collagen has higher swelling capacity compared to the remaining structure, which may be due to the larger collagen diameter size. As shown in Fig. 4g, the wide-spaced collagen shows a swell bubbling structure losing its original periodic feature. The *in situ* stiffness map (Fig. 4h) indicates a sharp contrast to distinguish the swollen wide-spaced collagen out of the remaining structure, in which the dark blue colour indicates the softer wide-spaced collagen. The stiffness distribution shows that the stiffness of the wide-spaced collagen is 1.0 ± 0.7 MPa while the stiffness of the remaining structure is 2.0 ± 0.7 MPa. Compared the mechanical properties of the DMs under ambient condition with that under physiological buffer condition, it demonstrates that precise maintenance of hydration is a vital factor for DMs biophysical property. This result is consistent with our previous study of the cornea stroma^35^. Since there is dysfunction of ECs in FECD, the barrier function and pump function are disturbed, which may alter the structure and mechanical property of DM.

## Conclusion

The ultrastructure and mechanical properties of FECD-DMs have been studied by TEM and QD-AFM. The results show that FECD alters the composition of DM with increased deposition of different collagens on the posterior surface, leading to thickening of the DM complex. Three kinds of different FECD-DMs are found in this study. The FECD-DMs are much thicker than the control ones. The short fibrillar type VIII collagen has been identified to comprise a large part of the abnormally secreted posterior collagenous layer of DM, where it forms the endothelial guttae characteristic of this disorder. The micro-periodicity of the fusiform wide-spaced bundles is approximately 120 nm. Moreover, the structure and mechanical properties of FECD-DMs studied by QD-AFM show that the structural changes do not only occur at the guttae but also the whole surface of DM; The mechanical property study showed that the stiffness of the wide-spaced collagen is softer than the remaining structure both under dehydrated and fully hydrated condition. In addition, the swelling capacity of the wide-spaced collagen is different from the surrounding structures. Precise maintenance of the hydration of the cornea is critical for the mechanical properties of DM. These findings may help for both clinical and material scientists to further understand the pathophysiological mechanisms of FECD and may improve future ways of diagnosis and treatment of the disease.

## Methods

### Samples preparation

The FECD-DM samples were from corneal transplant surgery, which replaced the cornea by donated corneal tissue (the graft) in its entirety (penetrating keratoplasty) or in part (lamellar keratoplasty), supplied by Aarhus University Hospital, Denmark. All surgeries were performed according to the guidelines for the Department of Ophthalmology, Aarhus University Hospital. The control samples are from the donated corneal tissue. The DM tissues were obtained and used for scientific purpose and ethical issues have been handled according to Danish healthcare law, and after guidance from the local ethical committee and national health authorities, and in accordance with the Declaration of Helsinki. Informed consent was obtained from all subjects. After corneal transplant surgery, the FECD-DM samples were kept in the 0.9% NaCl solution and were further used in the AFM characterization. All experimental protocols were approved by the Department of Ophthalmology, Aarhus University Hospital.

### TEM Analysis

The samples are fixed in 2% glutaraldehyd and 0.1 M cacodylate solution immediately after it is removed from the patient. The samples remained in this solution from 1 hour up to several days. For postfix where used 1% OsO_4_ in 0.1 M cacodylate (pH 7.2, 1 h) and 0.5% uranylacetat in 0.05 M maleat buffer (1 h), the samples were further dehydrated in different concentration of ethanol (from 70% to 99%). The dehydrated samples were infiltrated into the isopropanole and epon (1:1) over night and infiltrated into the pure epon for another 8 h. The imbedding samples were polymerized at 60 °C for 48 h. The blocks were cut on a Ultramikrotome Leica Ultracut UCT (Leica Mikrosysteme GmbH, Hernalser Hauptstrasse 219, Vienna, A-1170, Austria) with a 45° diamond knife from Diatome (Diatome, AG P.O. Box, 2501 Biel/Switzerland). The normal thickness of the cut samples is about 40–60 nm. Section placed on 300-mesh nickel grid, covered with carbon-coated formvar. After stained by uranylacetate and leadcitrate solution, the samples were observed in a transmission electron microscopy (CM 100, FEI) operating at 80 kV. Images are recorded by CCD camera 1K MegaView III (Olympus Soft Imaging Solutions GmbH, Johann-Krane-Weg 39, 48149 Münster) Image handling by AnalySiS from same Company.

### AFM measurement

The AFM images and quantitative mechanical measurements were recorded with Peakforce Tapping mode on a commercial Nanoscope VIII MultiMode SPM system (Bruker, Santa Barbara, CA) under both ambient (temperature, 24 **°**C; humidity, 42%) and physiological buffer (0.9% NaCl solution) conditions. For the ambient measurements, the DM samples were deposited onto the freshly cleaved mica surface, air-dried. The silicon tips (rectangular, MPP-13120-10, recommended stiffness measurement range: 1 GPa < E < 20 GPa, Bruker) with a spring constant of 209.3 N/m and a normal tip radius of 10.8 nm was used for mechanical measurement. AFM imaging was performed at a scan frequency of 0.5 Hz with optimized feedback parameters and 512- × 512-pixel resolution. For the liquid measurements, the DM samples were immersed in the physiological buffer while scanning. The silicon tips (Triangular, SCANASYST-FLUID, recommended stiffness measurement range: 1 MPa < E < 20 MPa, Bruker) with a spring constant of 0.72 N/m and a normal tip radius of 20 nm were used for morphology and mechanical measurement for hydrated samples. The measurement was performed at a scan frequency of 0.4 Hz with optimized parameters and 512- × 512-pixel resolution. The spring constants and tip radius of each AFM tip were calibrated before experiments according to Brucker’s application note.

### Data Analysis

The Young’s modulus reflects the stiffness of the DM, which was fitted by the Sneddon mode, where a rigid cone is punched into the soft tissue surface. The Young’s modulus therefore can be calculated as following:


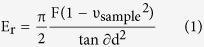



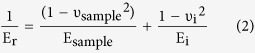


where 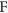 is the loading force; 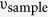 is the passion ratio of the sample; 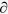 is the tip half cone opening angle and 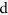 is the indentation; 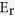 is the reduced Young’s modulus of the sample; 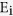 and 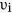 are the Young’s modulus and passion ratio of the tips. In our case of penetrating hard tips into soft DM tissues, 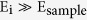, thus the second term of equation (2) is neglected. As FECD-DM and normal DM mainly consist of collagen fibers, we consider them as the materials, similar to polymer, and the 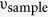 was estimated as 0.3 in the final data fitting. So the Young’s modulus of the samples can be deducted from the equations. In the AFM measurement system, Young’s modulus is obtained by fitting the linear part of the retrace force-distance curves. All the images and size distribution were analysed by using the commercial software Scanning Probe Image Processor. All force curves were analysed with offline software NanoScope Analysis (Bruker, Santa Barbara, CA).

## Additional Information

**How to cite this article**: Xia, D. *et al*. The Ultrastructures and Mechanical Properties of the Descement’s Membrane in Fuchs Endothelial Corneal Dystrophy. *Sci. Rep*. **6**, 23096; doi: 10.1038/srep23096 (2016).

## Supplementary Material

Supplementary Information
