# Supplementary material for: The Ultrastructures and Mechanical Properties of the Descement’s Membrane in Fuchs Endothelial Corneal Dystrophy
Source: Sci Rep. 2016 Mar 16;6:23096. doi: 10.1038/srep23096 (PMC4793225; doi:10.1038/srep23096)
Supplement: Supplementary Information [file srep23096-s1.doc]

Supporting Information

**The ultrastructures and mechanical properties of the Descement’s membrane in Fuchs endothelium corneal dystrophy**

Dan Xia^1,2^, Shuai Zhang^2^, Esben Nielsen^3^, Anders Ramløv Ivarsen^3^, Chunyong Liang^1^, Qiang Li^2^, Karen Thomsen^2^, Jesper Østergaard Hjortdal^3^, Mingdong Dong^2^*

^1^Research Institute for Energy Equipment Materials, Tianjin Key Laboratory of Materials Laminating Fabrication and Interface Control Technology, Hebei University of Technology, Tianjin 300130, China

^2^The Interdisciplinary Nanoscience Center, Aarhus University, Aarhus 8000, Denmark

^3^Department of Ophthalmology, Aarhus University Hospital, Aarhus 8000, Denmark

Correspondence and requests for materials should be addressed to M. D. (email: dong@inano.au.dk)


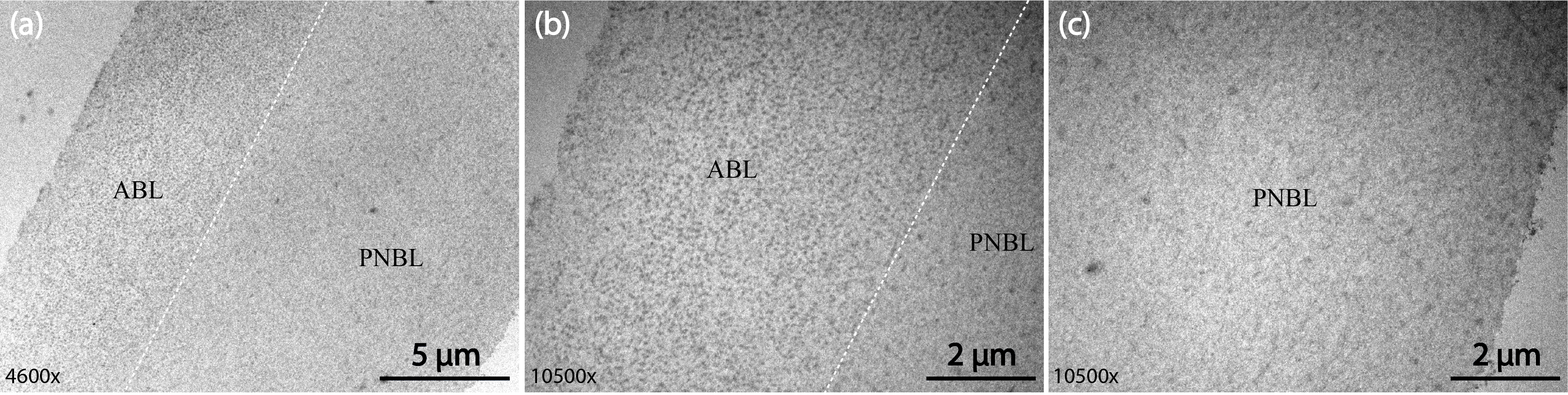


Figure S1 TEM image of the control DM sample (a), where the ABL and PNBL are clearly seen; TEM images of the

ABL (b) and the PNBL (c) zoomed in from (a).


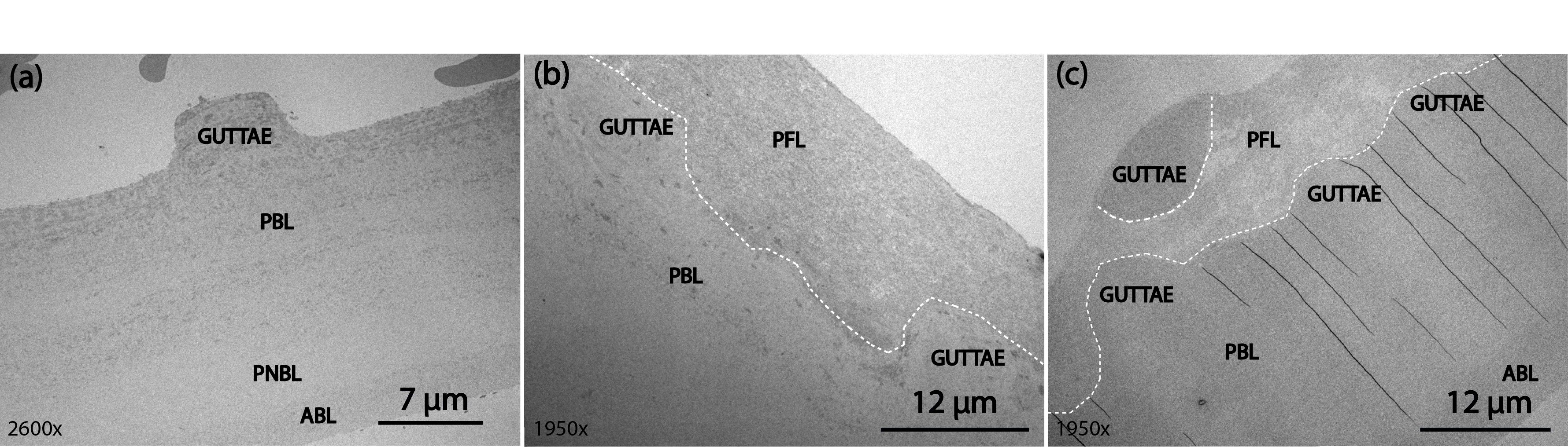


Figure S2 The overview image of type I (a), type II (b) and type III (c) FECD-DMs. The white dash lines indicate the boundary between the PFL and the guttae.


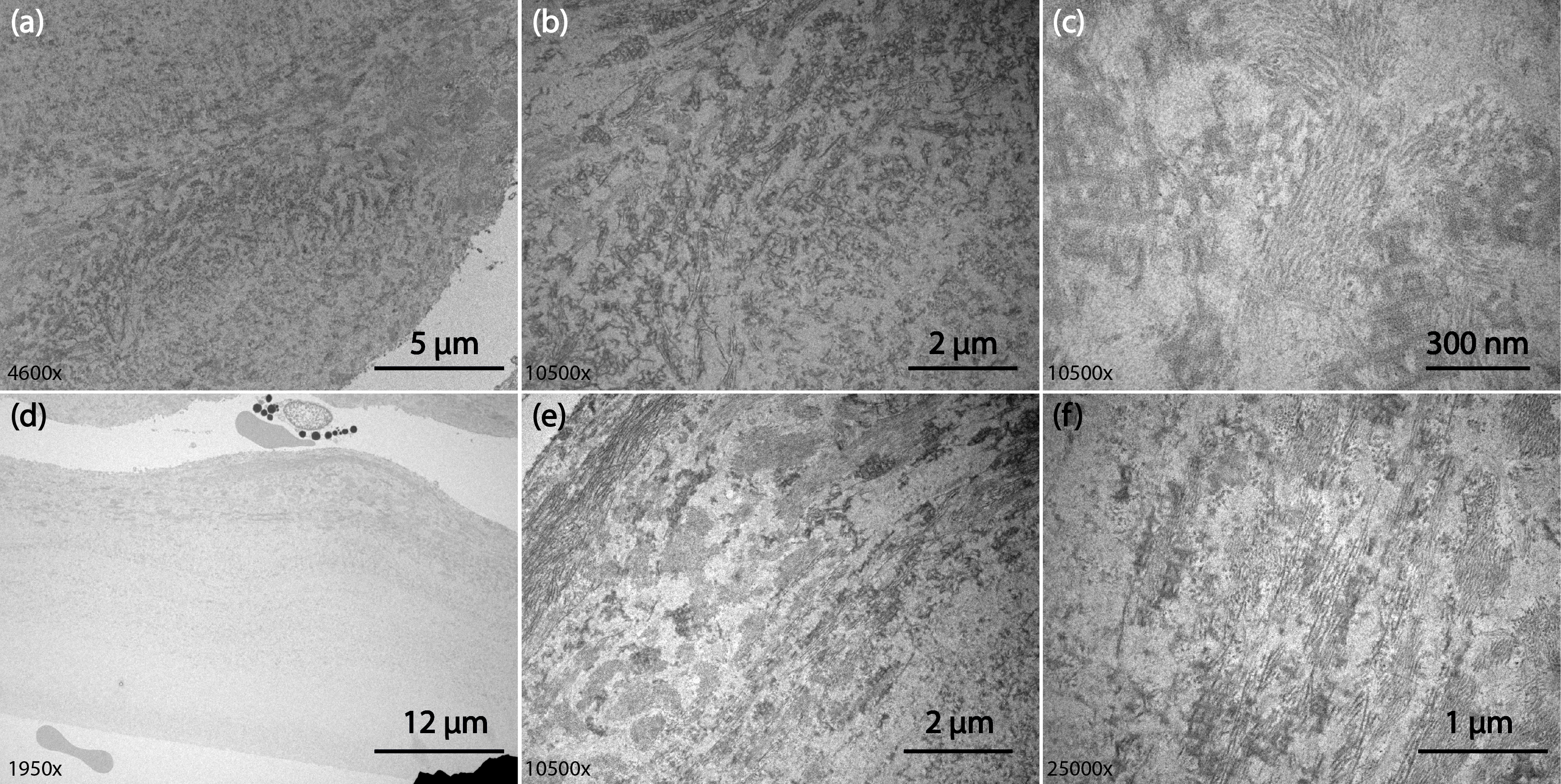


Figure S3 TEM images of two DMs with different guttae sizes; (a-c) The high magnification TEM images of Figure 1e in the main text; (d-f) Another FECD-DM with smaller guttae size.


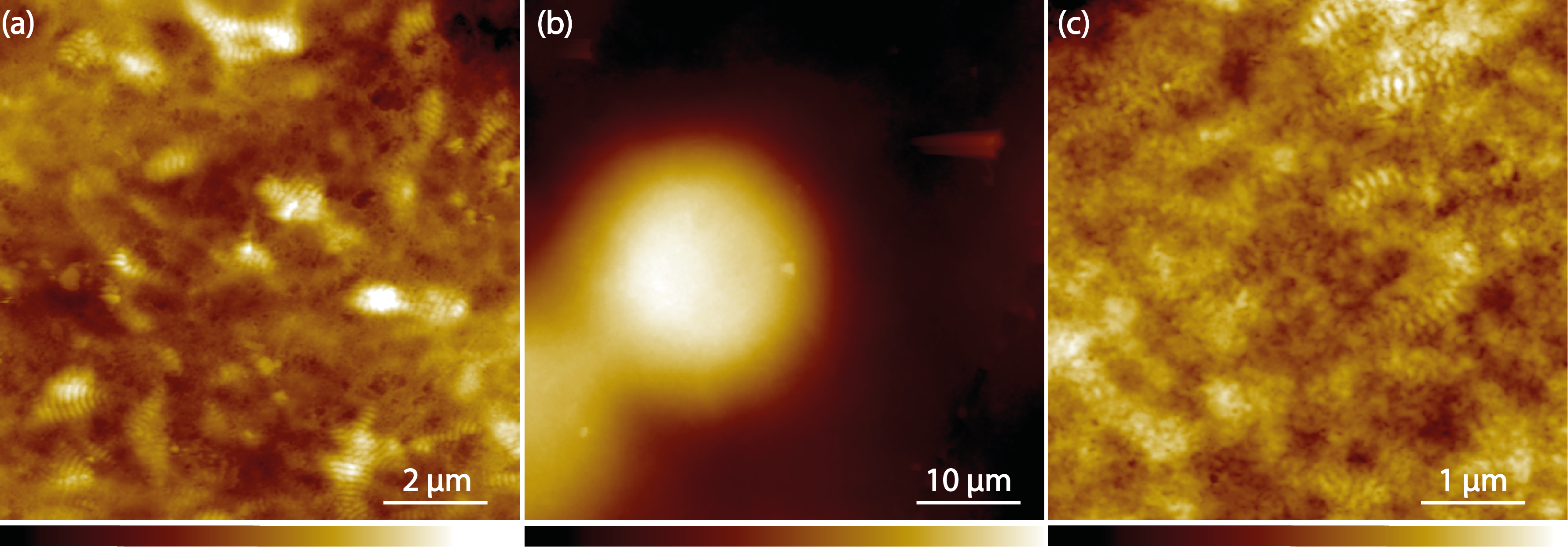


Figure S4 (a) The morphology of the FECD-DM surface far away from guttae; (b) The overview AFM image of a guttae on the FECD-DM surface; (c) The zoomed in AFM image from (b) on the guttae. The Z ranges of (a) and (c) are 0 nm to 170.5 nm while that of (b) is from 0 μm to 2.2 μm.
